# Development of Poly(l-Lactic Acid)-Based Bending Actuators

**DOI:** 10.3390/polym12051187

**Published:** 2020-05-22

**Authors:** Daniela M. Correia, Liliana C. Fernandes, Bárbara D.D. Cruz, Gabriela Botelho, Verónica de Zea Bermudez, Senentxu Lanceros-Méndez

**Affiliations:** 1 CQ-VR, University of Trás-os-Montes e Alto Douro, 5000-801 Vila Real, Portugal; 2 Centre of Physics, University of Minho, 4710-057 Braga, Portugal; lilianafernandes1411@gmail.com; 3 Centre of Chemistry, University of Minho, 4710-057 Braga, Portugal; barbara.cruz5@hotmail.com (B.D.D.C.); gbotelho@quimica.uminho.pt (G.B.); 4 Department of Chemistry, University of Trás-os-Montes e Alto Douro, 5000-801 Vila Real, Portugal; 5 BCMaterials, Basque Center for Materials, Applications and Nanostructures, UPV/EHU Science Park, 48940 Leioa, Spain; senentxu.lanceros@bcmaterials.net; 6 Ikerbasque, Basque Foundation for Science, 48013 Bilbao, Spain

**Keywords:** PLLA, ionic liquid, composites, degree of crystallinity, thermal treatments

## Abstract

This work reports on the development of bending actuators based on poly(l-lactic acid) (PLLA)/ionic liquid (IL) blends, through the incorporation of 40% wt. of the 1-ethyl-methylimidazolium bis(trifluoromethylsulfonyl)imide ([Emim][TFSI]) IL. The films, obtained by solvent casting at room temperature and 50 °C, were subjected to several post-thermal treatments at 70, 90, 120 and 140 °C, in order to modify the crystallinity of the films. The influence of the drying temperature and of [Emim][TFSI] blending on the morphological, structural, mechanical and electrical properties of the composite materials were studied. The IL induced the formation of a porous surface independently of the processing conditions. Moreover, the [Emim][TFSI] dopant and the post-thermal treatments at 70 °C promoted an increase of the degree of crystallinity of the samples. No significant changes were observed in the degree of crystallinity and Young Modulus for samples with thermal treatment between 70 and 140 °C. The viability of the developed high ionic conductive blends for applications as soft actuators was evaluated. A maximum displacement of 1.7 mm was achieved with the PLLA/[Emim][TFSI] composite prepared at 50 °C and thermally treated at 140 °C, for an applied voltage of 10 Vpp, at a frequency of 100 mHz. This work highlights interesting avenues for the use of PLLA in the field of actuators.

## 1. Introduction

Poly(l-lactic acid) (PLLA) is a biodegradable and biocompatible thermoplastic polymer, which exhibits a wide variety of interesting features [1], including piezoelectricity [2]. On account of its wide versatility, PLLA has been extensively explored for the development of biomaterials targeting several biomedical applications, such as drug delivery [3,4] and tissue engineering [5,6,7]. Further, its potential for the fields of biosensors and actuators has been demonstrated [2,8,9].

Another very attractive feature offered by PLLA is the possibility of adjusting its degree of crystallinity by thermal annealing treatments and/or upon introduction of dopants, such as ionic liquids (ILs), which allow controlling its complex highly ordered structure, which is composed of intermingled crystalline and amorphous regions [2,9]. 

Typically, ILs are defined as salts composed solely by organic cations and organic or inorganic anions, with a melting point below 100 °C. In recent years, due to their tunable properties and technological impact, such as high ionic conductivity [10], and high thermal, chemical and electrochemical stability [11,12,13], as well as low vapor pressure and volatility [14,15], ILs have been successfully employed in various domains for the design of numerous materials with enhanced features [16]. Additionally, because of their benign nature, ILs may play the role of green solvents with enormous potential in the field of sustainable chemistry [17,18]. 

ILs have been incorporated into several host polymer matrices for the development of polymer/IL materials targeting a myriad of applications [16], including actuators [16,19,20,21]. The influence of different ILs, such as 1-butyl-3-methylimidazolium dibutylphosphate [22], 3-methyl-1-(ethoxycarbonylethyl)imidazolium tetrafluoroborate [23] and 1-butyl-3-methylimidazolium hexafluorophosphate [24] on the physical-chemical properties of PLLA were reported. However, to the best of our knowledge, the possibility of using PLLA/IL based materials in actuators has never been addressed so far. 

In this work, PLLA and PLLA/IL films comprising the 1-ethyl-methylimidazolium bis(trifluoromethylsulfonyl)imide ([Emim][TFSI]) IL were developed by solvent casting to be applied as actuators. The films were prepared at different drying temperatures (25 and 50 °C), and subsequently subjected to four different post-thermal treatments in order to tune the degree of crystallinity, and, therefore, actuator performance. Their morphological, structural, mechanical, and electrical properties were characterized, and the influence of the post-thermal treatments investigated. Finally, the performance of the PLLA/IL composites as actuators was evaluated in terms of the influence of the drying temperature and post-thermal treatment temperatures on their bending response.

## 2. Materials and Methods

### 2.1. Materials

PLLA (Lumini L175, Corbion Purac, Montmeló, Spain; molecular weight (Mw): 217.000–225.000 g mol^−1^, melting temperature: 175 °C, glass transition: 55–60 °C, tensile modulus: 3500 MPa; tensile strength: 50 MPa), 1-ethyl-methylimidazolium bis(trifluoromethylsulfonyl)imide ([Emim][TFSI], 99%, Iolitec, Heilbronn, Germany) and dichloromethane (DCM, 99%, Merck, Darmstadt, Germany) were used as received. 

### 2.2. Preparation of the Neat and Composite PLLA-Based Films

**Figure 1:**
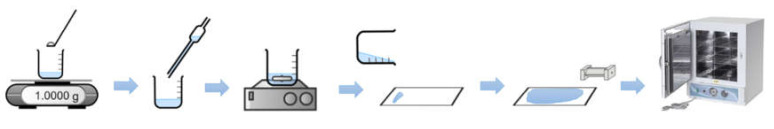
Schematic representation of the procedure used in the processing of the poly(l-lactic acid) (PLLA) and PLLA/[Emim][TFSI] films.

PLLA and PLLA/[Emim][TFSI] films were prepared using the solvent casting technique. To obtain the neat films, 1 g of PLLA was dissolved in 6.04 mL of DCM under magnetic stirring until complete dissolution. The solution was then cast onto a glass substrate and the solvent was evaporated (boiling temperature of DCM ≈ 40 °C), either at room temperature (DT25, where DT stands for drying treatment) overnight, or at 50 °C (DT50) for 30 min (Figure 1). In the case of the preparation of the PLLA/[Emim][TFSI] films (40 wt% of IL), the IL was mixed with 6.04 mL of DCM. The [Emim][TFSI] was selected, attending to its good miscibility with the DMC and its electrical conductivity (6.63 mS/cm, data obtained from the provider). Moreover, a concentration of 40 wt% was used attending to the commonly maximum IL concentration used in IL/polymer blends for actuator applications, as reported in our previous studies [25]. Then, PLLA was added and after its complete dissolution, the resulting solution was spread onto a glass substrate and left to dry, either at room temperature or at 50 °C in an oven. The PLLA and PLLA/[Emim][TFSI] films were then subjected to different thermal treatments (TT) of 70, 90, 120 and 140 °C for 30 min [26]. These samples will be henceforth named TT 70, TT 90, TT 120 and TT 140, respectively. Neat PLLA and PLLA/[Emim][TFSI] films (DT25 and DT50) with a thickness of ~40 µm and ~62 µm were obtained (see Supplementary Information Table S1). 

### 2.3. Characterization

#### 2.3.1. Morphological, Structural and Thermal Analyses

The samples morphology was evaluated using a Scanning Electron Microscopy (SEM) in a NanoSEM—FEI Nova 200 (FEG/SEM) (FEI, Hillsboro, OR, USA), with an accelerating voltage of 10 kV. Prior to the analysis the samples were coated with gold (Au) by magnetron sputtering (Polaron SC502). The attenuated total reflection (ATR)/Fourier transform infrared spectroscopy (FTIR) spectra were recorded with a Jasco FT/IR-4100 FTIR (Jasco, Pfungstadt, Germany) spectrometer, at room temperature in the 4000 and 600 cm^−1^ range, using 64 scans and a resolution of 4 cm^−1^. DSC measurements were performed using a DSC 200 F3 Maia Netzsch calorimeter from NETZSCH Premier Technologies (Iphofen, Germany), between 30 and 220 °C at a heating rate of 10 °C min^−1^ in a nitrogen atmosphere. For the measurements, approximately 4 mg of each sample was used. The degree of crystallinity (*X_c_*) was calculated using Equation (1):

(1) $$X_{c}\;\left(\%\right)=\frac{\Delta H}{\Delta H^{o}m}\times 100$$

where ΔH is the melting enthalpy, and $\Delta H^{o}m\;$is the melting enthalpy, for a fully crystallized PLLA sample (93.1 J g^−1^ [26]). The mechanical measurements were carried out in the tensile mode at room temperature in triplicate for each processing condition, in a universal testing machine (model AG-IS), from Shimadzu (Kyoto, Japan), using a load cell of 50 N. The samples, with a rectangular shape (30 mm × 10 mm), were analyzed at a speed of 1 mm min^−1^.

#### 2.3.2. Electrical and Electromechanical Characterization

The electrical measurements were performed with a Keithley 6430 (Allied, Las Vegas, NV, USA) picoammeter/voltage source. Prior to the experiments, the samples circular Au electrodes of 5 mm diameter were deposited in parallel in both sides of the samples by magnetron sputtering (Polaron Coater SC502, Quorum, Lewes, UK), thus ensuring a good electric contact between the sample and sample holder. The volume d.c. electrical conductivity was obtained at room temperature from the characteristic I-V curves, where the current (I) and voltage (V) were measured between −3 and +3 V and the conductivity (σ) was calculated considering the geometrical characteristics of the samples according to Equation (2):

(2) σ=1RAL

where *R* is the electrical resistance, *L* is the sample thickness and *A* is the area of the electrodes. The performance of the films as actuators was evaluated by bending tests in a home-made samples holder [25]. Prior to the measurements, the samples with dimensions of 12 mm × 2 mm were covered with Au on both sides by magnetron sputtering (Polaron SC502). The needles of the sample holder, clamping the bottom of the samples, were connected to an Agilent 33220A (Allied, Las Vegas, NV, USA) function generator. The displacement (δ) of the sample tip was measured by applying a square wave signal with peak-to-peak voltages (Vpp) of 5 and 10 V, at a frequency of 100 mHz.

## 3. Results and Discussion

### 3.1. Morphology

**Figure 2:**
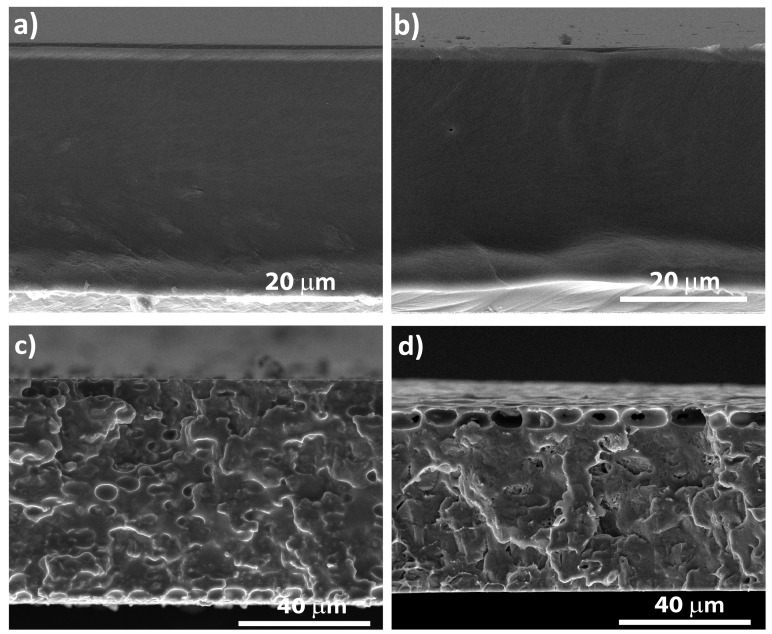
Cross-section SEM images of the PLLA-based films: (**a**) PLLA-DT25, (**b**) PLLA-DT50, (**c**) PLLA/[Emim][TFSI]-DT25 and (**d**) PLLA/[Emim][TFSI]-DT50.

The morphology of the PLLA and PLLA/[Emim][TFSI] films obtained at a room temperature and at 50 °C was evaluated by SEM. The cross-section images of the films are presented in Figure 2. 

Independently of the solvent evaporation temperature, the PLLA films exhibited a homogenous and non-porous texture, as shown in the cross-section SEM images (Figure 2a,b). Figure 2c,d demonstrate that upon [Emim][TFSI] incorporation into the PLLA matrix, and independently of the drying temperature, the IL induced the occurrence of a significant roughness and porosity. This effect was reported previously for other polymeric matrices (e.g., poly(vinylidene fluoride) (PVDF) [27]), and was attributed to the strong [Emim][TFSI]-DCM interaction, phase separation within the polymer solutions and [Emim][TFSI] trapping within the pores, once the solvent is evaporated from the solvent+IL rich regions [28]. 

### 3.2. Physical-Chemical Characterization

**Figure 3:**
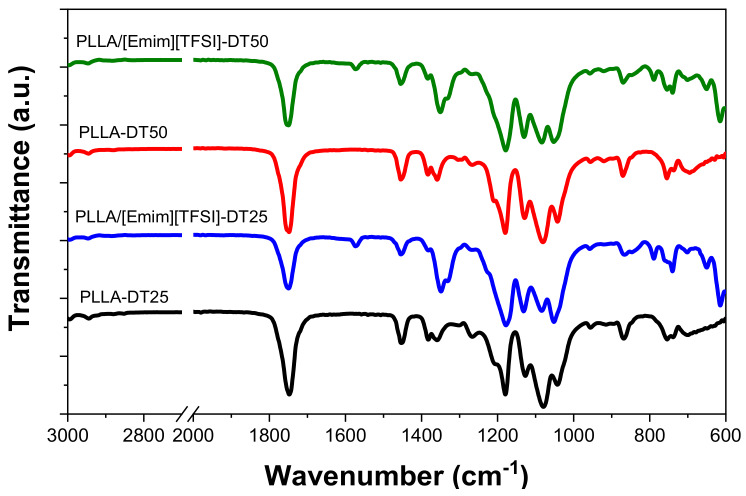
ATR/FTIR spectra of the PLLA and PLLA/[Emim][TFSI] films obtained at different drying temperatures.

To evaluate possible chemical modifications occurring in the PLLA polymer structure depending on the film processing at different drying temperatures, and to determine the effect of blending PLLA with [Emim][TFSI], all samples were analyzed by ATR/FTIR spectroscopy. The ATR/FTIR spectra are reproduced in Figure 3. The assignment of the main vibration bands is given in Table 1. 

The ATR/FTIR spectra of the PPLA-DT25 and PLLA-DT50 films display the main characteristic absorption bands of PLLA. No relevant differences are observed, depending on the processing temperature. The bands observed at 865 and 754 cm^−1^ are attributed to the stretching vibrations of the C-C(=O)O and C=O moieties of the ester group, respectively [29,30]. The absorption band detected at 955 cm^−1^ is assigned to the C–C and CH_3_ stretching vibrations. The absorption band at 1080 cm^−1^ corresponds to the asymmetric stretching of the C–O–C vibration. Finally, the most relevant bands between 2999 and 1750 cm^−1^ corresponds to the stretching vibrations of the CH_3_ and C=O groups, respectively [30,31].

The main absorption bands of pristine PLLA are also found in the ATR/FT-IR spectra of the PLLA/[Emim][TFSI] blends. These spectra also include the characteristic absorption bands of the IL at 1570 and 610 cm^−1^ assigned to the vibration of the N–CH_3_ and N–CH_2_–CH_3_ groups of the imidazolium ring, and to the CF_3_ groups of the TFSI^-^ anions, respectively. The intensity increase of the band at 1350 cm^−1^ is indicative of the overlapping of the absorption bands from both components of the blend [32]. 

**Figure 4:**
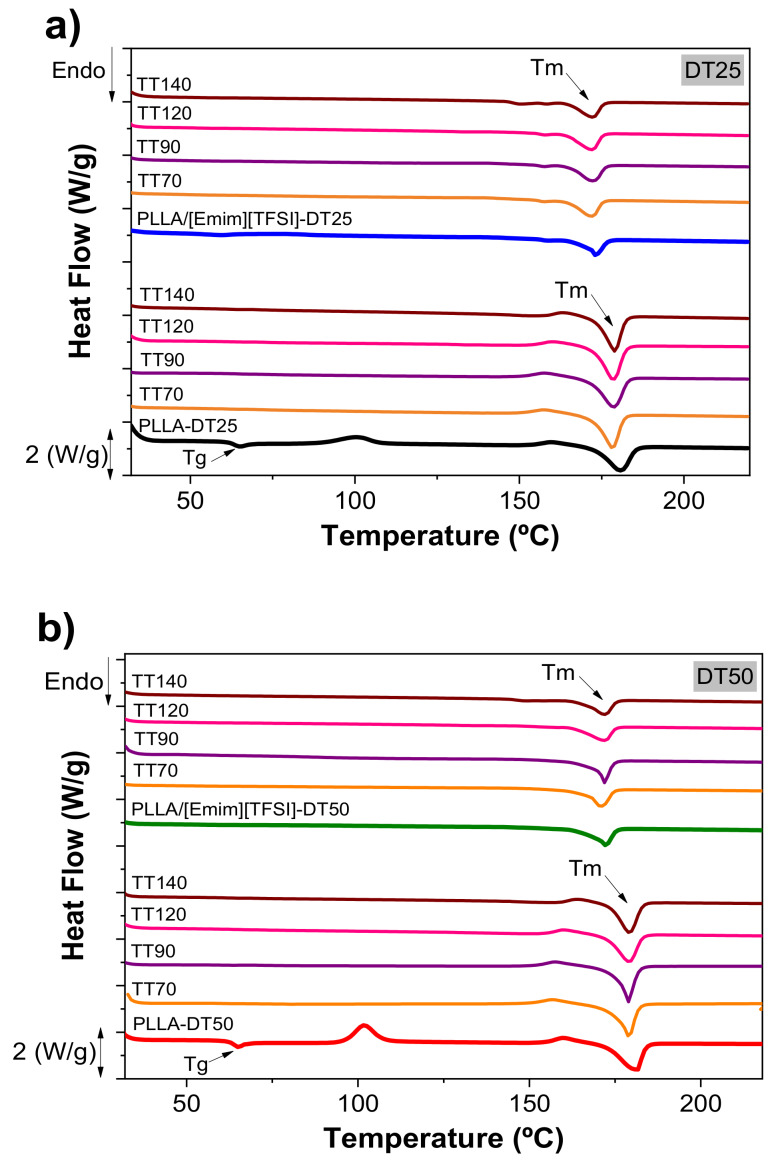
DSC curves as a function of the post-thermal treatment temperature for the PLLA-based films dried at: (**a**) 25 °C and (**b**) 50 °C.

The influence of the [Emim][TFSI] incorporation and the effect of the drying treatment on the thermal properties and degree of crystallinity of PLLA and [Emim][TFSI] films was evaluated by DSC. Figure 4 shows the DSC curves obtained for all the samples before and after the drying treatment. 

Both pristine PLLA samples, i.e., PPLA-DT25 and PPLA-DT50, produced endothermic peaks at ~67 and ~172 °C, respectively (Figure 4a,b), associated with the PLLA glass transition (T_g_) and melting (T_m_) temperatures, respectively [30,31]. The exothermic peak appearing between 100 and 160 °C is related to the polymer cold crystallization [30,31]. 

Upon addition of [Emim][TFSI] to the PLLA matrix, and independently of the drying temperature, two effects are evident. Figure 4a,b demonstrate that the events attributed to the glass transition and cold crystallization disappeared. In contrast, no significant changes were observed in the T_m_ value of the polymer. 

The influence of the post-thermal treatment performed on the degree of crystallinity was further assessed. Figure 4 allows inferring that a slight decrease in the T_m_ value of PLLA was observed for the post-thermal treatment carried out at 70 °C. The use of higher post-thermal treatment temperatures did not cause, however, any significant changes in T_m_. 

**Figure 5:**
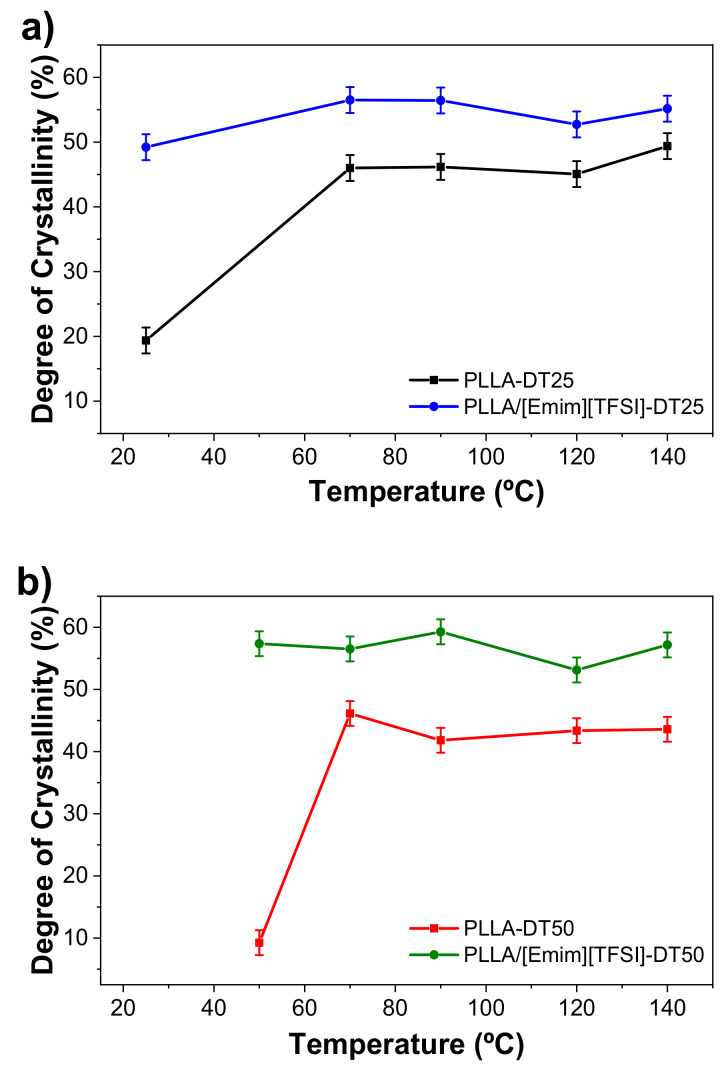
Degree of crystallinity as a function of the post-thermal treatment temperature for the PLLA-based films dried at: (**a**) 25 °C; and (**b**) 50 °C.

The effect of [Emim][TFSI] blending and post-thermal treatments on the degree of crystallinity were quantitatively evaluated by means of Equation (1), on the basis of the DSC data. The results are shown in Figure 5 and collected in Table S1. 

Table S1 allows inferring that a significant increase of the degree of crystallinity from ca. 19 to 49% resulted upon IL incorporation for the samples dried at 25 °C. Similarly, an increase of crystallinity was also observed for the samples dried at 50 °C (from ca. 9 to 57%). The similar trend observed for both composites at different drying temperatures indicates that the IL acts as a nucleating agent, inducing the formation of a higher number of nucleation centers accelerating the crystallization process [33]. This influence of the IL in the crystallization process of a polymer matrix has been reported for related polymer/IL blends [34].

Likewise, the post-thermal treatments promoted an important increase in the degree of crystallinity of the samples (Table S1). In all cases, this increase was more marked for the post-thermal treatment performed at 70 °C: from 19% to 46% and from 49% to 56% in the case of PLLA-DT25 and PLLA/[Emim][TFSI]-DT25, respectively. For the samples dried at 50 °C (PLLA-DT50 and PLLA/[Emim][TFSI]-DT50), an increase also resulted, but the maximum values of the degree of crystallinity remained practically the same as those of the samples dried at room temperature. In contrast, the increase of the post-thermal treatment temperature from 70 to 140 °C does not promote relevant variations in the degree of crystallinity, obtaining values of 46% and 44%, respectively. 

The highest degree of crystallinity values is observed in the polymer blends, as a result of the higher number of nucleation centers induced by the presence of the IL. Upon heating, the growth of nucleation centers led to a higher degree of crystallinity [26]. 

### 3.3. Mechanical Properties

**Figure 6:**
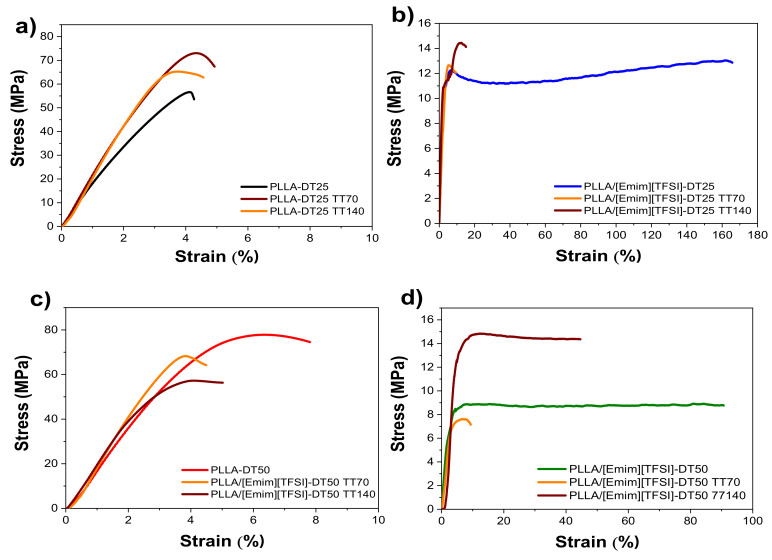
Stress vs. strain curves of PLLA and PLLA/[Emim][TFSI] films dried at 25 °C (**a** and **b**) and 50 °C (**c** and **d**), for samples subjected to post-thermal treatment at 70 and 140 °C.

The mechanical properties of the samples were determined by uniaxial stress-strain measurements. The goal was to evaluate the effect of the drying treatment, [Emim][TFSI] addition, and post-thermal treatments on the Young modulus (*E*). The results of the tensile stress measurements for PLLA and the PLLA/[Emim][TFSI] films dried at 25 and 50 °C, before and after the post-thermal treatments at 70 and 140 °C, are reproduced in Figure 6. The *E* values of all the samples are given in Table S2. 

Figure 6 demonstrates that, independently of the drying temperature, the PLLA films exhibited behavior typical of a thermoplastic polymer [35]. The [Emim][TFSI] added to the PLLA matrix exerted a plasticizing effect manifested in a noticeable elastic deformation region, followed by yielding and a linear regime. Additionally, it is also observed that, independently of the drying temperature, the IL incorporation into the PLLA matrix induces an increase in the elongation at break and ductility. The nominal *E* value was deduced from the linear regime of the elastic region and using the tangent method (Figure 6 and Table S2). 

Figure 6 and Table S2 allow one to conclude that the drying temperature slightly influenced the *E* value of the samples, increasing from 1410 ± 340 MPa (PLLA-DT25) to 1820 ± 280 MPa (PLLA-DT50). For the PLLA/[Emim][TFSI] films, a marked decrease of the *E* value occurred in PLLA/[Emim][TFSI]-DT25 (390 ± 160 MPa) and PLLA/[Emim][TFSI]-DT50 (380 ± 80 MPa). This decrease may be correlated with the plasticizing effect of [Emim][TFSI] [20,25]. For the PLLA samples dried at room temperature, no relevant changes are observed in *E* value when the post-thermal treatment temperature was increased from 70 °C (1840 ± 330 MPa) to 140 °C (2000 ± 100 MPa). However, for samples post-treated at 90 and 120 °C, a slight decrease of *E* is observed, being more noticeable for the samples post-thermal treated at 120 °C. This fact is associated to the slight decrease of the degree of crystallinity, as shown in Table S2, decreasing the stiffness of the samples. 

**Figure 7:**
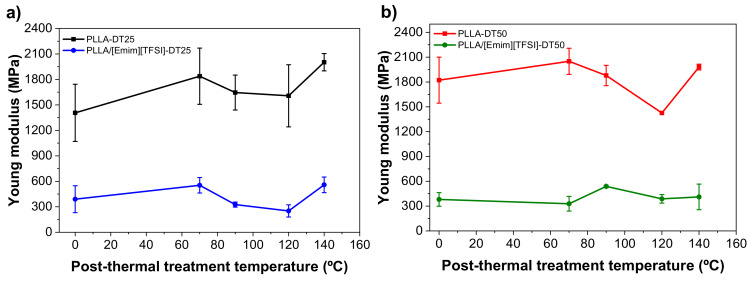
Young modulus as a function of the post-thermal treatment temperature for PLLA and PLLA/[Emim][TFSI] films dried at: (**a**) 25 °C and (**b**) 50 °C.

In the case of the PLLA/[Emim][TFSI]-DT25 sample, a small increase from 390 ± 160 MPa (without post-treatment) to 550 ± 90 MPa (PLLA/[Emim][TFSI]-DT25-TT70) was followed by a decrease for PLLA/[Emim][TFSI]-DT25-TT90 and PLLA/[Emim][TFSI]-DT25-TT120. The PLLA/[Emim][TFSI] sample treated at 140 °C exhibited an *E* value of 560 ± 90 MPa. A similar trend was observed for the PLLA-DT50 and PLLA/[Emim][TFSI]-DT50 materials. The Young Modulus behavior for all the samples and the effect of thermal treatments are summarized in Figure 7 and Table S3. 

### 3.4. Electrical Properties

The influence of [Emim][TFSI] on the electrical properties of the PLLA/[Emim][TFSI] composites was evaluated. Additionally, the influence of the post-thermal treatments was also investigated for the samples post-treated with the minimum and maximum temperatures (70 and 140 °C). 

**Figure 8:**
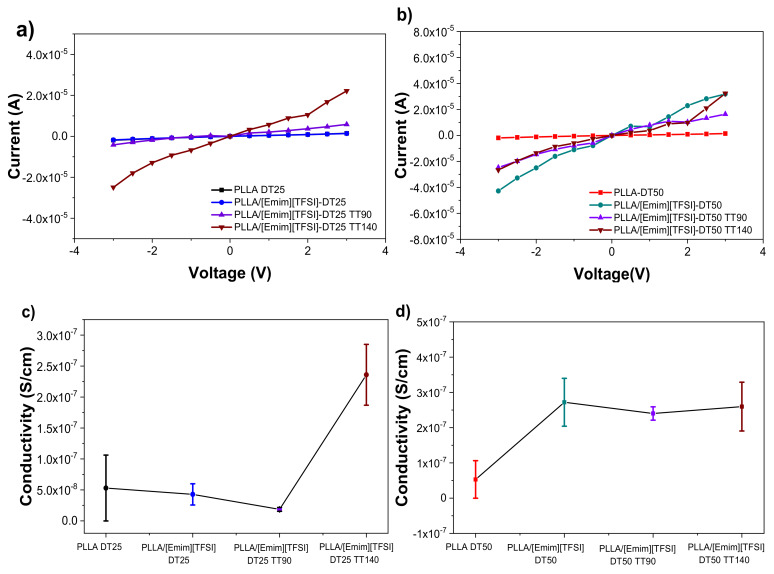
IV curves (**a**,**b**) and electrical conductivity (**c**,**d**) as a function of the post-thermal treatment temperature of the PLLA-based films dried at 25 °C (left column) and 50 °C (right column).

Figure 8a,b show that the samples dried at 25 and 50 °C evidenced, as expected, a linear relation between I and V. The electrical conductivity of the samples was evaluated using Equation (2) and the results are reproduced in Figure 8c,d. 

In the case of the samples dried at room temperature, the conductivity remained practically the same (5.30 × 10^−8^ to 4.28 × 10^−8^ S cm^−1^ for PPLA-DT25 and PLLA/[Emim][TFSI]-DT25, respectively). The post-thermal treatment at 90 °C led to a slight decrease to 1.86 × 10^−8^ S cm^−1^ for the PLLA/[Emim][TFSI]-DT25. However, the electrical conductivity of [Emim][TFSI]/PLLA-DT25TT140 increased substantially to 2.36 × 10^−5^ S/m, probably because at this temperature (close to the T_m_ of PLLA), the energy supplied induced the optimal orientation of the polymeric chains, promoting ion transport.

In the case of the samples dried at 50 °C (Figure 8d), a significant increase in the electrical conductivity from 5.30 × 10^−8^ to 2.72 × 10^−7^ S cm^−1^ resulted from the addition of the [Emim][TFSI] to the host matrix [20]. The post-thermal treatments performed at 90 and 140 °C exerted no significant changes in the electrical conductivity. Furthermore, one should notice that no significant changes occur in the electrical conductivity of [Emim][TFSI]/PLLA-DT50 before and after the post-thermal treatments. These results are associated to the similar degree of crystallinity of the samples for a given number of charge carriers, in which the amorphous phase plays a determinant role into the ionic carriers transport [20,36].

### 3.5. Electromechanical Measurements

**Figure 9:**
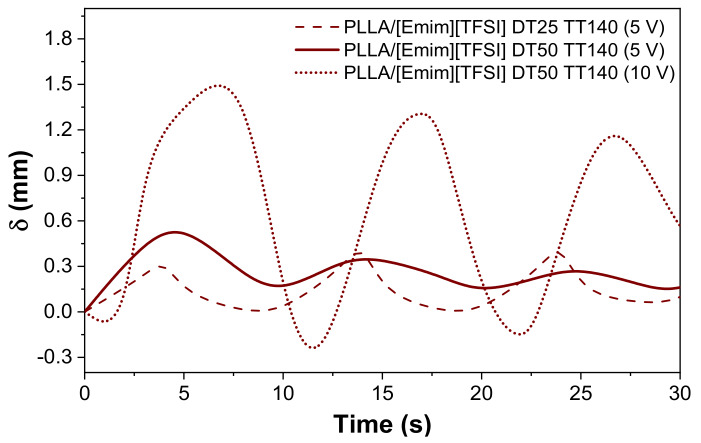
Displacement as a function of time for the PLLA/[Emim][TFSI]-DT25 and PLLA/[Emim][TFSI]-DT50 samples, subjected to a post-thermal treatment at 140 °C.

The potential of the developed composites for the development of biocompatible soft actuators was evaluated by electromechanical measurements. Considering the high electrical conductivity measured for PLLA/[Emim][TFSI]-DT25 and PLLA/[Emim][TFSI]-DT50 post-treated at 140 °C, the electromechanical measurements were focused exclusively on these samples. The displacement as a function of time upon an applied voltage of 5 and 10 Vpp at a frequency of 100 mHz is represented in Figure 9. 

**Figure 10:**
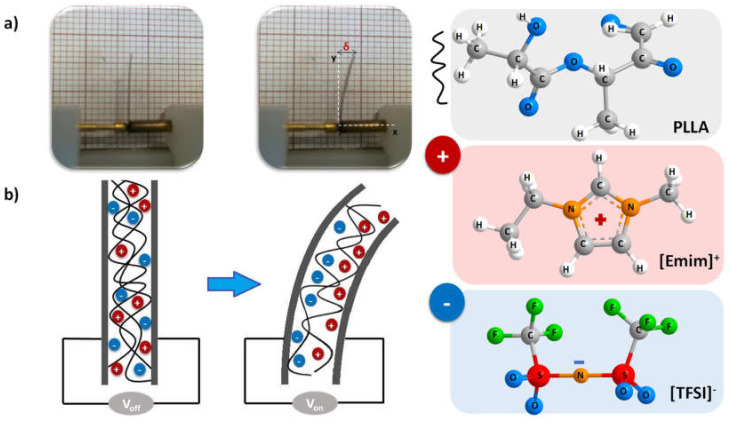
(**a**) Displacement observed for the PLLA/[Emim][TFSI]-DT25 TT140, upon an applied voltage of 10 Vpp at a frequency of 100 mHz; (**b**) Schematic representation of the ionic movement caused by an applied voltage.

The observed displacements (δ) were measured from the position of the actuator tip upon an applied voltage of 5 Vpp at a frequency of 100 mHz. Figure 9 shows that for an applied voltage of 5 Vpp, the PLLA/[Emim][TFSI]-DT25 film exhibited a maximum displacement ranging from ~0.3 to 0.4 mm. The maximum displacement was observed for the PLLA/[Emim][TFSI]-DT50 composite, with values ranging between 0.2 and 0.5 mm. For these samples, with the increase in the applied voltage from 5 to 10 Vpp, an increase in the displacement to 1.7 mm resulted (Figure 10a), indicating that high voltages favored the ions’ movement within the polymer matrix [19]. The strain developed as a response to the applied electrical field resulted from the diffusion of the ions and migration to the positive (cations) and negative (anions) electrode layers, leading to the migration of the cations and anions, and subsequent accumulation close to the electrodes [19,37] (Figure 10b). 

It must be also noticed that the displacement does not follow a symmetric behavior, which is manifested by the non-symmetric displacement curves with respect to the initial position, as a result of the irreversible movement of ions and relaxation. Upon voltage application, due to the different cation and anion sizes, an imbalance of the ion transport occurs, leading to a non-symmetrical ions dynamical behavior (Figure 10b) [19].

## 4. Conclusions

The PLLA polymer and PLLA/[Emim][TFSI] composites were obtained by solvent casting at different drying temperatures (25 and 50 °C). Additionally, the films were subjected to different post-thermal treatments, to study their influence on the degree of crystallization of pristine and composite films. The effect of the drying temperature on the morphological and chemical properties was evaluated. Independently of the drying temperature, the incorporation of [Emim][TFSI] into the PLLA matrix promoted the formation of a porous texture. The degree off crystallinity increased with [Emim][TFSI] addition, and with post-thermal treatments performed at 70 °C. No significant changes occurred with the increase of the post-thermal treatment temperature from 70 to 140 °C. With respect to mechanical measurements, [Emim][TFSI] acted as a plasticizer, decreasing the Young Modulus. No significative changes are observed in the Young modulus for the samples dried at room temperature and post-thermal treated at temperatures ranging from 70 °C to 140 °C. Electrical conductivity measurements revealed an increase in the conductivity values with [Emim][TFSI] blending and post-thermal treatments. We demonstrate here that the developed high ionic conductivity blends offer great potential as soft actuators. A maximum displacement of 1.7 mm was measured for the sample PLLA/[Emim][TFSI]-DT50-TT140 for an applied voltage of 10 Vpp, at a frequency of 100 mHz. 

## Figures and Tables

**Table 1:** Main absorption bands of PLLA and [Emim][TFSI] in the prepared samples.

Wavenumbers (cm^−1^)	Attribution	
	PLLA [29]	[Emim][TFSI] [32]
**2997**	CH_3_ asymmetric stretching	-
**1752**	C=O stretching	-
**1570**	-	ring vibration N–CH_3_ and N–CH_2_–CH_3_
**1454**	CH_3_ asymmetric stretching	-
**1383**	CH_3_ symmetric stretching	-
**1358**	CH stretching+CH_3_ symmetric stretching	-
**1348**		SO_2_ symmetric stretching
**1300**	CH stretching	-
**1184**	C–O–C +CH_3_ asymmetric stretching	CF_3_ antisymmetric stretching
**1132**	CH_3_ asymmetric stretching	SO_2_ symmetric stretching
**1085**	C–O–C symmetric stretching	-
**1045**	C–CH_3_ stretching	
**1051**		C–C and NCH_3_ stretching
**921**	C–C stretching + CH_3_ rotation	-
**871**	C–COO stretching	
**738**	-	CF_3_ stretching
**755**	C=O stretching	-
**740**		CF_3_ symmetric stretching
**651**		S–N–S bending
**619**	-	SO_2_ antisymmetric bending

## Supplementary Materials

The following are available online at https://www.mdpi.com/2073-4360/12/5/1187/s1, Table S1, Thickness of the PLLA and PLLA/IL films as a function of the drying and post-treatment temperature, Table S2: Degree of crystallinity of the PLLA and PLLA/[Emim][TFSI] films as a function of drying temperature and post-thermal treatment; Table S3: Young modulus for PLLA and PLLA/[Emim][TFSI] films.

## References

[1] Farah S., Anderson D.G., Langer R. (2016). Physical and mechanical properties of PLA, and their functions in widespread applications—A comprehensive review. Adv. Drug Deliv. Rev..

[2] Chorsi M.T., Curry E.J., Chorsi H.T., Das R., Baroody J., Purohit P.K., Ilies H., Nguyen T.D. (2019). Piezoelectric Biomaterials for Sensors and Actuators. Adv. Mater..

[3] Tyler B., Gullotti D., Mangraviti A., Utsuki T., Brem H. (2016). Polylactic acid (PLA) controlled delivery carriers for biomedical applications. Adv. Drug Deliv. Rev..

[4] Hyon S.H. (2000). Biodegradable poly (lactic acid) microspheres for drug delivery systems. Yonsei Med. J..

[5] Yang F., Murugan R., Ramakrishna S., Wang X., Ma Y.X., Wang S. (2004). Fabrication of nano-structured porous PLLA scaffold intended for nerve tissue engineering. Biomaterials.

[6] Ribeiro C., Sencadas V., Correia D.M., Lanceros-Méndez S. (2015). Piezoelectric polymers as biomaterials for tissue engineering applications. Colloids Surf. B Biointerfaces.

[7] Correia D.M., Sencadas V., Ribeiro C., Martins P.M., Martins P., Gama F.M., Botelho G., Lanceros-Méndez S. (2016). Processing and size range separation of pristine and magnetic poly(l-lactic acid) based microspheres for biomedical applications. J. Colloid Interface Sci..

[8] Curry E.J., Ke K., Chorsi M.T., Wrobel K.S., Miller A.N., Patel A., Kim I., Feng J., Yue L., Wu Q. (2018). Biodegradable Piezoelectric Force Sensor. Proc. Natl. Acad. Sci. USA.

[9] Tajitsu Y. (2013). Fundamental study on improvement of piezoelectricity of poly(ι-lactic acid) and its application to film actuators. IEEE Trans. Ultrason. Ferroelectr. Freq. Control.

[10] Noshadi I., Walker B.W., Portillo-Lara R., Shirzaei Sani E., Gomes N., Aziziyan M.R., Annabi N. (2017). Engineering Biodegradable and Biocompatible Bio-ionic Liquid Conjugated Hydrogels with Tunable Conductivity and Mechanical Properties. Sci. Rep..

[11] Ye Y.-S., Rick J., Hwang B.-J. (2013). Ionic liquid polymer electrolytes. J. Mater. Chem. A.

[12] Dong K., Liu X., Dong H., Zhang X., Zhang S. (2017). Multiscale Studies on Ionic Liquids. Chem. Rev..

[13] Zhang S.G., Zhang Q.H., Zhang Y., Chen Z.J., Watanabe M., Deng Y.Q. (2016). Beyond solvents and electrolytes: Ionic liquids-based advanced functional materials. Prog. Mater. Sci..

[14] Lei Z., Chen B., Koo Y.-M., MacFarlane D.R. (2017). Introduction: Ionic Liquids. Chem. Rev..

[15] Xu P., Cui Z.-P., Ruan G., Ding Y.-S. (2019). Enhanced Crystallization Kinetics of PLLA by Ethoxycarbonyl Ionic Liquid Modified Graphene. Chin. J. Polym. Sci..

[16] Correia D.M., Fernandes L.C., Martins P.M., García-Astrain C., Costa C.M., Reguera J., Lanceros-Méndez S. (2020). Ionic Liquid–Polymer Composites: A New Platform for Multifunctional Applications. Adv. Funct. Mater..

[17] Yoo C.G., Pu Y.Q., Ragauskas A.J. (2017). Ionic liquids: Promising green solvents for lignocellulosic biomass utilization. Curr. Opin. Green Sustain. Chem..

[18] Marr P.C., Marr A.C. (2016). Ionic liquid gel materials: applications in green and sustainable chemistry. Green Chem..

[19] Mejri R., Dias J.C., Hentati S.B., Botelho G., Esperanca J., Costa C.M., Lanceros-Mendez S. (2016). Imidazolium-based ionic liquid type dependence of the bending response of polymer actuators. Eur. Polym. J..

[20] Dias J.C., Correia D.M., Costa C.M., Ribeiro C., Maceiras A., Vilas J.L., Botelho G., de Zea Bermudez V., Lanceros-Mendez S. (2019). Improved response of ionic liquid-based bending actuators by tailored interaction with the polar fluorinated polymer matrix. Electrochim. Acta.

[21] Terasawa N. (2018). High-performance transparent actuator made from Poly(dimethylsiloxane)/Ionic liquid gel. Sens. Actuators B-Chem..

[22] Wei T., Pang S., Xu N., Pan L., Zhang Z., Xu R., Ma N., Lin Q. (2015). Crystallization behavior and isothermal crystallization kinetics of PLLA blended with ionic liquid, 1-butyl-3-methylimidazolium dibutylphosphate. J. Appl. Polym. Sci..

[23] Gui H., Li Y., Chen S., Xu P., Zheng B., Ding Y. (2014). Effects of biodegradable imidazolium-based ionic liquid with ester group on the structure and properties of PLLA. Macromol. Res..

[24] Gardella L., Furfaro D., Galimberti M., Monticelli O. (2015). On the development of a facile approach based on the use of ionic liquids: preparation of PLLA (sc-PLA)/high surface area nano-graphite systems. Green Chem..

[25] Correia D.M., Barbosa J.C., Costa C.M., Reis P.M., Esperança J.M.S.S., de Zea Bermudez V., Lanceros-Méndez S. (2019). Ionic Liquid Cation Size-Dependent Electromechanical Response of Ionic Liquid/Poly(vinylidene fluoride)-Based Soft Actuators. J. Phys. Chem. C.

[26] Ribeiro C., Sencadas V., Costa C.M., Gómez Ribelles J.L., Lanceros-Méndez S. (2011). Tailoring the morphology and crystallinity of poly(L-lactide acid) electrospun membranes. Sci. Technol. Adv. Mater..

[27] Mazumder M., Ahmed R., Wajahat Ali A., Lee S.-J. (2018). SEM and ESEM techniques used for analysis of asphalt binder and mixture: A state of the art review. Constr. Build. Mater..

[28] Ribeiro C., Costa C.M., Correia D.M., Nunes-Pereira J., Oliveira J., Martins P., Gonçalves R., Cardoso V.F., Lanceros-Méndez S. (2018). Electroactive poly(vinylidene fluoride)-based structures for advanced applications. Nat. Protoc..

[29] Kawai T., Rahman N., Matsuba G., Nishida K., Kanaya T., Nakano M., Okamoto H., Kawada J., Usuki A., Honma N. (2007). Crystallization and Melting Behavior of Poly (l-lactic Acid). Macromolecules.

[30] Krikorian V., Pochan D.J. (2005). Crystallization Behavior of Poly(l-lactic acid) Nanocomposites:  Nucleation and Growth Probed by Infrared Spectroscopy. Macromolecules.

[31] Cao X., Mohamed A., Gordon S.H., Willett J.L., Sessa D.J. (2003). DSC study of biodegradable poly(lactic acid) and poly(hydroxy ester ether) blends. Thermochim. Acta.

[32] Kiefer J., Fries J., Leipertz A. (2007). Experimental Vibrational Study of Imidazolium-Based Ionic Liquids: Raman and Infrared Spectra of 1-Ethyl-3-methylimidazolium Bis(Trifluoromethylsulfonyl)imide and 1-Ethyl-3-methylimidazolium Ethylsulfate. Appl. Spectrosc..

[33] Jiao Q., Chen Q., Wang L., Chen H.L., Li Y.J. (2019). Investigation on the Crystallization Behaviors of Polyoxymethylene with a Small Amount of Ionic Liquid. Nanomaterials.

[34] Correia D.M., Costa C.M., Lizundia E., Sabater i Serra R., Gómez-Tejedor J.A., Biosca L.T., Meseguer-Dueñas J.M., Gomez Ribelles J.L., Lanceros-Méndez S. (2019). Influence of Cation and Anion Type on the Formation of the Electroactive β-Phase and Thermal and Dynamic Mechanical Properties of Poly(vinylidene fluoride)/Ionic Liquids Blends. J. Phys. Chem. C.

[35] Mirkhalaf S.M., Fagerström M. (2019). The mechanical behavior of polylactic acid (PLA) films: fabrication, experiments and modelling. Mech. Time-Depend. Mater..

[36] Aziz S.B., Woo T.J., Kadir M.F.Z., Ahmed H.M. (2018). A conceptual review on polymer electrolytes and ion transport models. J. Sci. Adv. Mater. Devices.

[37] Reizabal A., Correia D.M., Costa C.M., Perez-Alvarez L., Vilas-Vilela J.L., Lanceros-Méndez S. (2019). Silk Fibroin Bending Actuators as an Approach Toward Natural Polymer Based Active Materials. ACS Appl. Mater. Interfaces.

