# Erratum: Development of Poly(l-Lactic Acid)-Based Bending Actuators. *Polymers* 2020, *12*, 1187

**DOI:** 10.3390/polym12112463

**Published:** 2020-10-23

**Authors:** Daniela M. Correia, Liliana C. Fernandes, Bárbara D. D. Cruz, Gabriela Botelho, Verónica de Zea Bermudez, Senentxu Lanceros-Méndez

**Affiliations:** 1 CQ-VR, University of Trás-os-Montes e Alto Douro, 5000-801 Vila Real, Portugal; 2 Centre of Physics, University of Minho, 4710-057 Braga, Portugal; lilianafernandes1411@gmail.com; 3 Centre of Chemistry, University of Minho, 4710-057 Braga, Portugal; barbara.cruz5@hotmail.com (B.D.D.C.); gbotelho@quimica.uminho.pt (G.B.); 4 Department of Chemistry, University of Trás-os-Montes e Alto Douro, 5000-801 Vila Real, Portugal; 5 BCMaterials, Basque Center for Materials, Applications and Nanostructures, UPV/EHU Science Park, 48940 Leioa, Spain; senentxu.lanceros@bcmaterials.net; 6 Ikerbasque, Basque Foundation for Science, 48013 Bilbao, Spain

The authors wish to make a change to their published paper [1]. In the original manuscript, there is a mistake in a sentence in Section 3.5, on page 10. Two words “anions and cations” were reverted by mistake. The corrected sentence is shown below:

The strain developed as a response to the applied electrical field results from the diffusion of the ions and migration to the positive (anions) and negative (cations) electrode layers, and subsequent accumulation close to the electrodes.

The authors apologize for any inconvenience caused, and the change does not affect the scientific results. The manuscript will be updated, and the original will remain online on the article webpage at https://www.mdpi.com/2073-4360/12/5/1187.
