# Supplementary material for: Development of Poly(l-Lactic Acid)-Based Bending Actuators
Source: Polymers (Basel). 2020 May 22;12(5):1187. doi: 10.3390/polym12051187 (PMC7285213; doi:10.3390/polym12051187)
Supplement: Supplementary file 1 [file polymers-12-01187-s001.pdf]

polymers

Supplementary information

Development of Poly(L-Lactic Acid)-Based Bending Actuators

Daniela M. Correia [1,2,*], Liliana C. Fernandes [2], Bárbara D.D. Cruz [3], Gabriela Botelho [3], Verónica de Zea Bermudez [1,4,*] and Senentxu Lanceros-Méndez [5,6]

1 CQ-VR, University of Trás-os-Montes e Alto Douro, 5000-801 Vila Real, Portugal
2 Centre of Physics, University of Minho, 4710-057 Braga, Portugal; lilianafernandes1411@gmail.com
3 Centre of Chemistry, University of Minho, 4710-057 Braga, Portugal; barbara.cruz5@hotmail.com (B.D.D.C.); gbotelho@quimica.uminho.pt (G.B.)
4 Department of Chemistry, University of Trás-os-Montes e Alto Douro, 5000-801 Vila Real, Portugal
5 BCMaterials, Basque Center for Materials, Applications and Nanostructures, UPV/EHU Science Park, 48940 Leioa, Spain; senentxu.lanceros@bcmaterials.net
6 Ikerbasque, Basque Foundation for Science, 48013 Bilbao, Spain
* Correspondence: d.correia@fisica.uminho.pt (D.M.C.); vbermude@utad.pt (V.D.Z.B.)

Table S1. Thickness of the PLLA and PLLA/IL films as a function of the drying and post-treatment temperature.

Post-Thermal Temperature (°C)	Thickness (µm)			
	Drying temperature: 25 °C		Drying temperature: 50 °C	
	PLLA	PLLA/[Emim][TFSI]	PLLA	PLLA/[Emim][TFSI]
No treatment	40 ± 1.15	67 ± 2.22	46±1.14	63±2.04
70	39 ± 0.37	65 ± 1.16	45±4.07	62±1.04
90	43 ± 0.37	60 ± 0.81	48±4.65	64±1.86
120	41 ± 0.74	64 ± 1.94	54±3.20	55±2.34
140	42 ± 0.82	63±1.17	43±1.03	59±1.33

Table S2. Degree of crystallinity of the PLLA and PLLA/[Emim][TFSI] films as a function of drying temperature and post-thermal treatment.

Post-Thermal Treatment (°C)	Degree of Crystallinity (%) (±2%)			
	Drying temperature: 25 °C		Drying temperature: 50 °C	
	PLLA	PLLA/[Emim][TFSI]	PLLA	PLLA/[Emim][TFSI]
No treatment	19	49	9.2	57
70	46	56	46	56
90	46	56	42	59
120	45	53	41	53
140	49	55	44	57

Table S3. Young modulus for PLLA and PLLA/[Emim][TFSI] films.

Drying temperature (°C)	Sample	Post-thermal treatment (°C)	Young modulus ± deviation (MPa)
25	PLLA	-	1410 ± 340
		70	1840 ± 330
		90	1650 ± 210
		120	1610 ± 370
		140	2000 ± 100
	PLLA/[Emim][TFSI]	-	390 ± 160
		70	550 ± 90
		90	330 ± 30
		120	250 ± 70
		140	560 ± 90
50	PLLA	-	1820 ± 280
		70	2050 ± 160
		90	1880 ± 120
		120	1430 ± 50
		140	1980 ± 30
	PLLA/[Emim][TFSI]	-	380 ± 80
		70	330 ± 90
		90	540 ± 20
		120	390 ± 50
		140	410 ± 0.15
